# Rivet-Inspired Modification of Aramid Fiber by Decorating with Silica Particles to Enhance the Interfacial Interaction and Mechanical Properties of Rubber Composites

**DOI:** 10.3390/ma13112665

**Published:** 2020-06-11

**Authors:** Yihang Li, Yuzhu Xiong, Qingpo Zhang

**Affiliations:** Department of Polymer Material and Engineering, College of Materials and Metallurgy, Guizhou University, Guiyang 550025, China; gs.liyh18@gzu.edu.cn

**Keywords:** surface modification, rivet-inspired method, hybrid structure, interfacial interaction, rubber composites

## Abstract

A rivet–inspired method of decorating aramid fiber (AF) with silica particles (SiO_2_) is proposed to produce SiO_2_@AF hybrid materials that have largely enhanced interfacial interaction with the rubber matrix. AF was firstly surface-modified with polyacrylic acid (PAA) to obtain PAA–AF, and SiO_2_ was silanized with 3-aminopropyltriethoxysilane to obtain APES–SiO_2_. Then, SiO_2_@AF was prepared by chemically bonding APES–SiO_2_ onto the surface of PAA–AF in the presence of dicyclohexylcarbodiimide (DCC) and 4-dimethylaminopyridine (DMAP). With the incorporation of SiO_2_@AF into the rubber matrix, SiO_2_@AF hybrid materials with high surface roughness can play a role as ‘rivets’ to immobilize large numbers of rubber chains on the surface. The tear strength and tensile strength of rubber composite that filling 4 phr SiO_2_@AF are dramatically increased by 97.8% and 89.3% compared to pure rubber, respectively. Furthermore, SiO_2_@AF has superiority in enhancing the cutting resistance of rubber composites, in contrast with unmodified AF and SiO_2_. SiO_2_@AF is suitable to be applied as a novel reinforcing filler in rubber composites for high performance.

## 1. Introduction

Natural rubber (NR), an important biopolymer, has been presented as one of the most attractive materials owing to its wonderful properties, such as great elasticity and flexibility, antivirus permeation, low cost and corrosion resistance [1,2,3]. However, NR usually needs to be reinforced with various fillers for most practical applications in diverse areas (e.g., tires, sealing material, sport equipment, surgical gloves and adhesives), due to its inherent low strength and modulus [4,5]. For decades, SiO_2_ particles have been widely applied as active fillers in rubber composites to enhance their mechanical properties and to deliver multi-functional characteristics [6,7]. Compared to traditional carbon black, SiO_2_ can effectively improve wet skid resistance and show higher abrasion resistance for tires, and is an ideal filler for the preparation of green tires [8,9]. At the same time, tires filled with SiO_2_ particles have lower rolling resistance, which has the great advantage of reducing vehicle energy consumption. Although SiO_2_ particles are extensively assumed as a superior substitute to common carbon black, inhibiting SiO_2_ particles from aggregating is extremely challenging because of their high surface energy and large surface area [10]. Heavily hydrophilic SiO_2_ particles exhibit a rather poor compatibility with hydrophobic rubber, which leads to weak interfacial adhesion between SiO_2_ and the rubber matrix. These devilish puzzles seriously impede the popularization and application of SiO_2_ fillers in rubber systems [11].

Currently, aramid fiber (AF) is an indispensable material in many fields and has become one of most excellent reinforcing fillers for high-performance composites due to its unique properties, for example its low specific density, thermal resistance, super fatigue, ultrahigh strength and modulus, and good chemical stability [12]. Aramid fiber reinforced rubber composite material combines the flexibility and high elasticity of rubber with the rigidity of fiber material, which has excellent mechanical properties, cutting resistance, tear resistance, puncture resistance and other properties [13,14,15]. Aramid fiber/rubber composite can be processed and formed by conventional rubber processing equipment like other rubber compounding agents. Aramid fiber reinforced rubber can reduce the weight and rolling resistance of tires, so as to prepare new composite materials with great potential for the development of ‘green tires’. However, AF commonly possesses a smooth and chemically inert surface, lacking functional groups, which leads to chemical and/or mechanical interlocking resistance to the polymer matrix [16]. Hence, it is difficult to acquire superior interfacial adhesion between virgin AF and the polymer matrix, heavily affecting the performance of its composites [17,18]. As described in many publications, numerous methods have been carried out to improve the interfacial interaction of AF with the polymer matrix, including plasma treatments [19], γ–ray irradiation [20], chemical etching [21], surface grafting [22] and direct fluorination [23]. However, chemical oxidation or etching damages the AF structures and lowers the strength to some extent. Furthermore, physical modification methods are usually performed under extremely rigorous conditions, leading to high costs [16]. Chemical grafting can only introduce a small number of functional groups on the AF surface, which limitedly improves the interfacial adhesion [22]. In addition, grafting a certain group onto the AF surface is only suitable for specific matrix materials, and a series of tedious and time-consuming pre-processes are commonly required for successful grafting [24].

Inspired by the rivet, depositing particles, for example SiO_2_ particles, onto the AF surface can greatly enhance the surface roughness and can effectively prevent AF from pulling out of the matrix. Therefore, depositing is an efficient fiber-modifying method with the advantages of fiber structures, repairing defects on the fiber surface and being compatible with different kinds of matrices [25]. Moreover, the decoration of SiO_2_ on the AF surface lends itself to overcoming the aggregation of individual particles. In previous reports, SiO_2_ particles have commonly been combined on the AF surface by the sol–gel process [22,26], or by the colloidal dispersion method. However, to the best of our knowledge, only a small number of nanoscale SiO_2_ particles are bonded onto the AF surface by the above-mentioned techniques.

**Figure 1:**
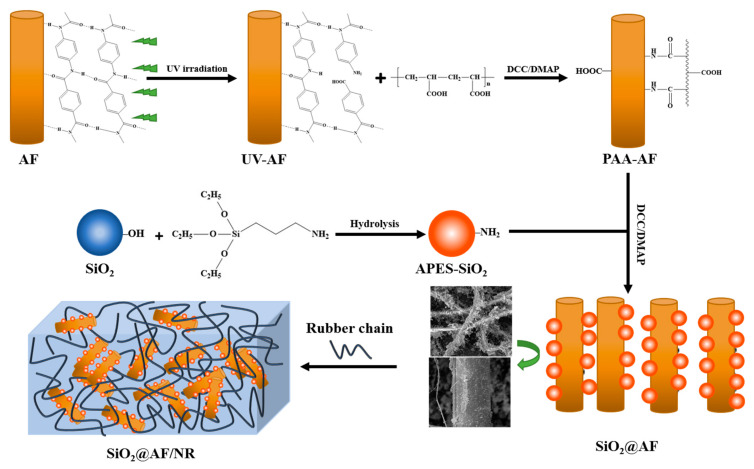
Schematic diagram of the preparation of the aramid fiber decorated with silica (SiO_2_@AF) hybrid materials and the natural rubber (NR) composites. AF, aramid fiber; SiO_2_, silica; DCC, dicyclohexylcarbodiimide; DMAP, 4-dimethylaminopyridine; PAA–AF, AF modified with polyacrylic acid; APES–SiO_2_, SiO_2_ modified by 3-aminopropyltriethoxysilane.

In this article, a mass of SiO_2_ macroparticles (48 wt%) were deposited on the AF surface by chemical bonding. A schematic diagram of preparing the AF decorated by SiO_2_ (SiO_2_@AF) is presented in Figure 1. Through our previous work and the reports of other scholars, it was found that the use of ultraviolet radiation can break the acylamino bond on the surface and can form new carboxyl and amino groups [27,28,29]. AF and SiO_2_ were modified with polyacrylic acid (PAA) and 3-aminopropyltriethoxysilane (APES), respectively. In sequence, SiO_2_ particles were adsorbed on the AF surface by the electrostatic interaction between the negatively charged carboxyl on the PAA grafting AF (PAA–AF) and the positively charged amino on the APES modified SiO_2_ particles (APES–SiO_2_). The amidation of carboxyl and amino can promote the combination of SiO_2_ particles with AF, due to the existence of dicyclohexylcarbodiimide (DCC) and 4-dimethylaminopyridine (DMAP). Then, the resultant SiO_2_@AF was incorporated into the rubber matrix to manufacture SiO_2_@AF/NR composites. SiO_2_@AF, with its high surface roughness, plays a role as a ‘rivet’ to better immobilize the NR chains on the filler surface. The interfacial adhesion between SiO_2_@AF and the NR matrix is greatly improved with the incremental fraction of immobilized chains, which results in the improvement of the mechanical properties and cutting resistance of SiO_2_@AF/NR composites.

## 2. Materials and Methods

### 2.1. Materials

Aramid fiber (AF; 1500 D, 1670 dtex) was purchased from DuPont (Wilmington, DE, USA) and cut to 3–5 cm in length before use. Silica (SiO_2_), with the trademark TS-180, was supplied by Changzhou Lehuan Chemical Co. Ltd (Changzhou, China). Polyacrylic acid (PAA), dicyclohexylcarbodiimide (DCC), 4-dimethylaminopyridine (DMAP), ethanol, tetrahydrofuran (THF) and 3-aminopropyltriethoxysilane (APES) of analytical grade were obtained from Shanghai Aladdin Biochemical Technology Company (Shanghai, China). The rubber ingredients, including zinc oxide (ZnO), stearic acid (SA), diphenyl hydrazine (D), 2-thiol benzothiazole (M), 2,6′-dithiodibenzothiazole (DM), tetramethylthiuram disulfide (TMTD), antioxidant 4010NA and sulfur (S) were of industrial grade and used without purification.

### 2.2. Grafting PAA onto AF

AF was ultrasonically cleaned in acetone solution and irradiated below a UV lamp for 8 min for pretreatment, with a relative humidity (RH) of 65 RH%. UV radiation can, in a short time, destroy the surface chemical structure of AF and can form new groups without affecting its core structure (Figures S1 and S2). AF 4.0 g, PAA 5.0 g, DCC 5.0 g and DMAP 0.5 g were dispersed into 500 mL THF under sonication for 1h. Then, the mixture was heated at 60 °C for 24 h to complete the amidation reaction. Finally, the product was filtered and washed with deionized water, and then dried in an oven at 80 °C until a constant weight was obtained. In this reaction, the number of carboxyl groups is excessive in order to remove the amino group from the surface of the AF.

### 2.3. Silanization of SiO_2_

First, 5.0 g SiO_2_ was added into 300 mL ethanol/deionized water (5:5, volume ratio) under sonication to achieve a well-dispersed SiO_2_ suspension. Subsequently, 2 mL APES was added into the SiO_2_ suspension. The mixture was stirred at 60 °C for 12 h to accomplish the silanization. The product (APES–SiO_2_) was filtrated, washed with ethanol and dried overnight at 60 °C in a vacuum oven.

### 2.4. Preparation of SiO_2_@AF

First, 4.0 g PAA–AF and 6.0 g APES–SiO_2_ were dispersed in 400 mL THF solution under sonication for 0.5 h. Then, the mixture was stirred mildly for another 0.5 h to perform the electrostatic assembly between the negatively charged PAA–AF and the positively charged APES–SiO_2_. Finally, 2.0 g DCC and 0.5 g DMAP were added, and the reaction was maintained at 60 °C for 48 h. In order to deposit as much SiO_2_ on the AF surface as possible, the amount of APES–SiO_2_ was excessive. Thus, SiO_2_@AF hybrid materials with 48 wt% SiO_2_ particles were obtained by drying at 60 °C overnight, after filtering the remaining APES–SiO_2_ and washing with deionized water.

### 2.5. Preparation of the Natural Rubber Composites

NR was compounded with the ingredients and SiO_2_@AF on a two–roll mixer at room temperature. The basic formulation of the NR composites is shown in Table 1. SiO_2_@AF/NR composites were vulcanized at 143 °C up to the optimum time, as determined by a Rubber Process Analyzer (RPA). Furthermore, SiO_2_+AF/NR composites (SiO_2_+AF represents the mixture filler of untreated SiO_2_ and AF with a weight ratio of 48:52) were prepared following a similar process. 

### 2.6. Characterization

Fourier transform infrared spectroscopy (FTIR) was recorded by a Thermo Nicolet 6700 FTIR spectrometer (Waltham, MA, USA) in the 4000–600 cm^−1^ region at room temperature. A resolution of 4 cm^−1^ and a total accumulation of 24 scans were applied to obtain the high signal-to-noise spectra. In order to analyze the chemical elements of PAA–AF, APES–SiO_2_ and SiO_2_@AF, X-ray photoelectron spectroscopic (XPS) analysis was conducted on an XPS analyzer (K-Alpha, Thermo, Waltham, MD, USA) with Al Kα radiation under an X-ray power of 150 W. Thermogravimetric analysis (TGA) was performed on a thermogravimetric analyzer (TA Q50, Newcastle, DE, USA) at a heating rate of 20 °C/min from 30 to 800 °C. Then, samples of 15 mg were isolated in a dry nitrogen atmosphere with a purge rate of 80 mL/min. The morphology of unmodified AF and SiO_2_@AF was observed by scanning electron microscope (SEM; JSM-7500F, JEOL, Tokyo, Japan) at an acceleration voltage of 10 kV. All of the fiber surfaces were sprayed with gold to prevent electrostatic charging. Differential scanning calorimetry (DSC) measurements were carried out on a DSC instrument (Q20, TA, Newcastle, DE, USA) with a nitrogen flow of 40 mL/min. Samples with a weight of 7–8 mg were scanned from 70 to 100 °C at a heating rate of 20 °C/min. The mechanical properties of the NR composites and pure NR were investigated using a universal testing machine (Hegewald & Peschke, Berlin, Germany). Specimens with a dumbbell shape were used for tensile characterizations according to GB/T 528–1998 at a crosshead speed of 500 mm/min. Tear tests were carried out with right-angle samples according to GB/T 529–1999. A cutting tester (RCC–1, Beijing Wanhui Yifang Science and Technology Development Company, Beijing, China) was utilized to analyze the cut resistance of the NR composites and the pure NR. The strip specimens were rolled at a speed of 720 cpm under a cutting frequency of 120 Hz for 20 min, and the loss of mass was recorded every 4 min. The weight loss was recorded as a function of cutting time in the measurement process. 

## 3. Results and Discussions

### 3.1. Characterizations of SiO_2_@AF

**Figure 2:**
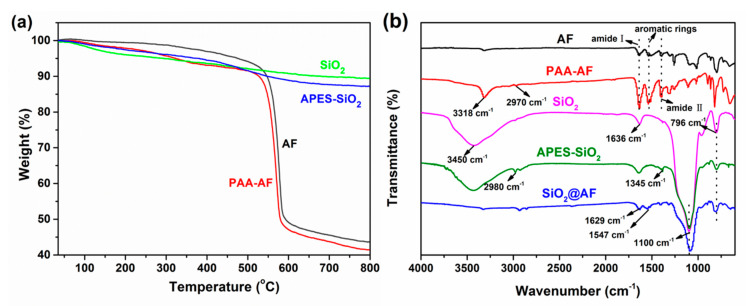
Thermogravimetric analysis (TGA) curves of SiO_2_, APES–SiO_2_, AF and PAA–AF (**a**). Fourier transform infrared spectroscopy (FTIR) spectra of AF, PAA–AF, SiO_2_, APES–SiO_2_ and SiO_2_@AF (**b**).

The grafting degree of PAA–AF and APES–SiO_2_ can be quantitatively evaluated by TGA. Figure 2a illustrates the TGA curves of neat SiO_2_, APES–SiO_2_, AF and PAA–AF. For all of the specimens, the weight loss at a temperature lower than 100 °C is mainly due to the evaporation of physically adsorbed water. The mass loss of pristine SiO_2_ exhibits another decomposition stage at 200–600 °C, which is attributed to the dehydration of the silanol groups [30]. In the TGA trace of AF, the sharp weight loss from approximately 500–600 °C represents the partial dihydroxylation and alkoxide decomposition of AF [31]. In addition, the PAA–AF fibers began to decompose at 180 °C due to the grafting of PAA (Figure 2a), and the weight loss between 500 and 800 °C further decreased to 41.4%. As shown in the TGA curves of APES–SiO_2_ and PAA–AF, it can be inferred that the grafting modification has little influence on the decomposition of the SiO_2_ or AF matrices. Thus, the weight loss percentage of the matrix (SiO_2_ or AF) at the temperature of 100–800 °C in both the untreated and treated samples is identical.

Taking AF as an example, the weight loss ratio (*ε*) of the aramid fiber matrix between 100 and 800 °C in PAA–AF is equal to that in pristine AF, which can be expressed as [32]

(1) $$\varepsilon =\frac{M_{A100}-M_{A800}}{M_{A100}}$$

where *M_A_*_100_ (99.8%) and *M_A_*_800_ (43.6%) represent the residual weight percentage of AF at 100 °C and 800 °C, respectively.

Considering that the grafting PAA is completely degraded during the heating process, the grafting content (*χ*) can be figured out with the help of Equation (2):

(2) $$\chi =\frac{(M_{P100}-M_{P800})-M_{P100}\times \varepsilon }{1-\varepsilon }÷M_{P100}$$

where *M_P_*_100_ (99.3 %) and *M_P_*_800_ (41.4%) stand for the residual weight percentage of PAA–AF at 100 °C and 800 °C, respectively. After calculation, the grafting content (*χ*) of PAA–AF is 4.56%. In addition, the grafting content of APES–SiO_2_ is calculated to be 3.07% by the same equations.

**Figure 3:**
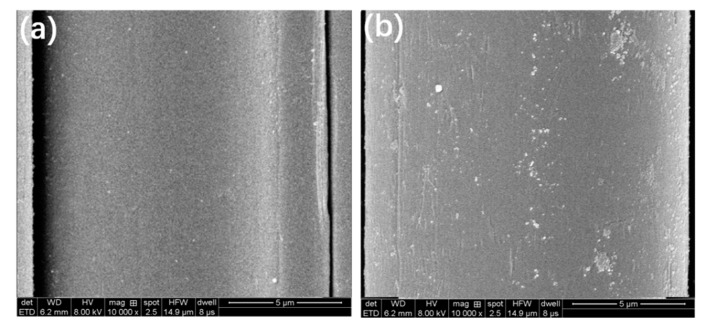
Scanning electron microscope (SEM) images of AF (**a**) and PAA–AF (**b**).

Figure 2b presents the FTIR spectra of AF, PAA–AF, SiO_2_, APES–SiO_2_ and SiO_2_@AF in the region of 600–4000 cm^−1^. As shown in Figure 2b, the C–N stretching peak at 1392 cm^−1^, N–H bending peak at 1548 cm^−1^ and C=O stretching peak at 1630 cm^−1^ can be found in the AF and PAA–AF spectra [33]. By contrast with common AF, the new absorption band assigned to –OH in the carboxyl groups is clearly observed at 3318 cm^−1^ in the FTIR spectrum of PAA–AF [34], which can be attributed to the carboxyl group from PAA. In addition, the peaks at 2970 cm^−1^ for the PAA–AF can be ascribed to the stretching vibrations of the methylene groups from PAA. Compared to AF, PAA–AF showed a broader and stronger band around 1548 cm^−1^, 1630 cm^−1^ and 1392 cm^−1^ (aromatic rings, amide I and amide П) due to the introduction of PAA by the acylamino bond [35]. Figure 3 shows the SEM image of AF and PAA–AF, and it clear that the surface AF is very smooth (Figure 3a), whereas the PAA–AF is obviously covered by a rough grafted layer (Figure 3b). Therefore, it is reasonable to conclude that PAA was successfully deposited on the AF surface.

In the SiO_2_ FTIR spectrum, the peaks observed at 3450 cm^−1^ and 1630 cm^−1^ are related to the stretching and bending vibrations of the hydroxyl groups from the hydrate water, respectively [36,37]. The absorption bands around 1100 cm^−1^ and 796 cm^−1^ are attributed to the asymmetric and symmetric stretching of Si–O–Si, respectively [38]. As shown in the APES–SiO_2_ FTIR graph, the bands at 2980 and 1345 cm^−1^ correspond to the stretching and bending vibrations of the methylene groups in siloxane, respectively [39]. This feature demonstrates that APES is indeed combined with SiO_2_. From the FTIR spectrum of SiO_2_@AF, the O–H stretching band disappears, which indicates that the–COOH grafted on PAA–AF is exhausted and the modified SiO_2_ is dehydrated during the hybridization process. However, the peak still observed at 1629 cm^−1^ is due to the stretching vibration of carbonyl in amide [40]. Another peak, which exists at 1547 cm^−1^, is ascribed to the N–H bending vibration of amide. This confirms that SiO_2_@AF was successfully prepared by the chemical bonding between APES–SiO_2_ and PAA–AF.

**Figure 4:**
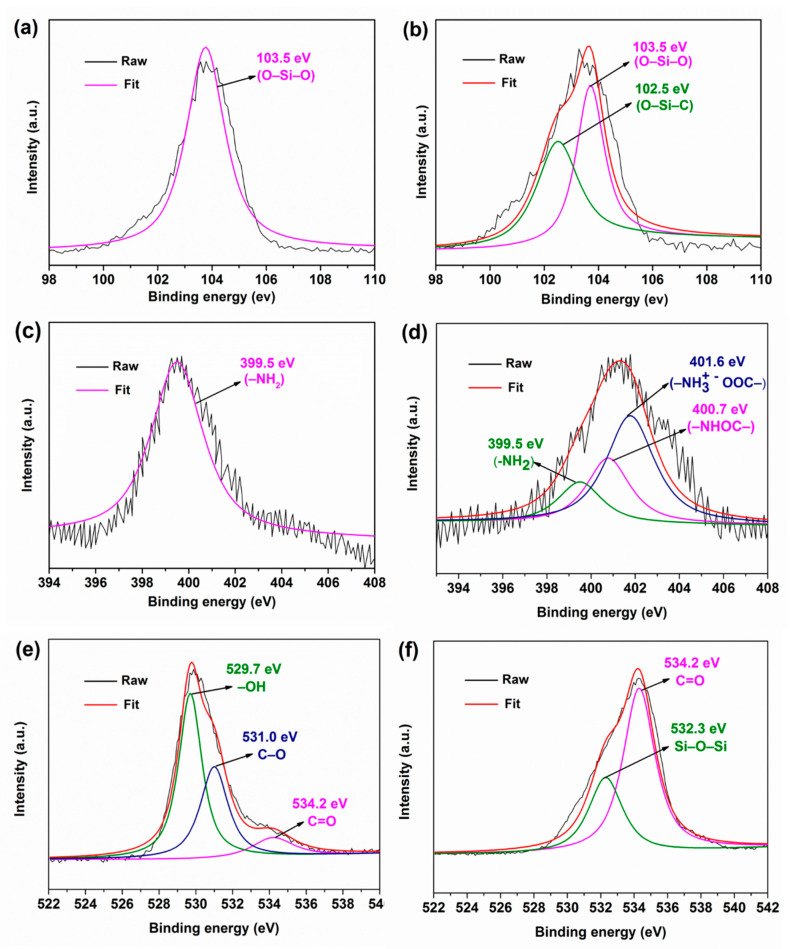
X–ray photoelectron spectroscopic (XPS) survey curves of (**a**) Si2p of SiO_2_, (**b**) Si2p of APES–SiO_2_, (**c**) N1s of APES–SiO_2_, (**d**) N1s of SiO_2_@AF, (**e**) O1s of PAA–AF and (**f**) O1s of SiO_2_@AF.

In order to further explore the combination of PAA–AF and APES–SiO_2_, XPS was applied to detect detailed any structural and compositional changes of the SiO_2_@AF samples. The XPS peaks fitted by the multipeak Lorentzian fitting program are presented in Figure 4. The Si2p core level spectrum of SO_2_ (Figure 4a) exhibits an obvious peak corresponding to O–Si–O at 103.5 eV. However, a new binding energy of the silicon atoms for APES–SiO_2_ (Figure 4b) is observed at 102.5 eV, which is assigned to the O–Si–C bond [41]. Figure 4c shows the N1s peak at 399.5 eV for APES–SiO_2_, which is attributed to nitrogen in amine. As displayed in Figure 4d, the N1s spectrum of SiO_2_@AF presents two new peaks at –NHOC– (400.7 eV) and –NH_3_^+^^–^OOC– (401.6 eV), which illustrates that SiO_2_ was combined with AF by covalent and ionic bonds [42]. The O1s peaks for PAA–AF observed at 529.7 eV, 531.0 eV and 534.2 eV in Figure 4e are ascribed to –OH, C–O, and C=O, respectively. Nevertheless, the –OH (529.7 eV) and C–O (531.0 eV) peaks disappear in the O1s curve of SiO_2_@AF (Figure 4f), which indicates that the –COOH grafted onto the AF was consumed during the preparation process. Another new peak at 532.3 eV, assigned to the oxygen atom (Si–O–Si), appears with the introduction of SiO_2_. All of these features confirm that the SiO_2_@AF hybrid materials were successfully obtained, which is consistent with the results of the FTIR analysis.

**Figure 5:**
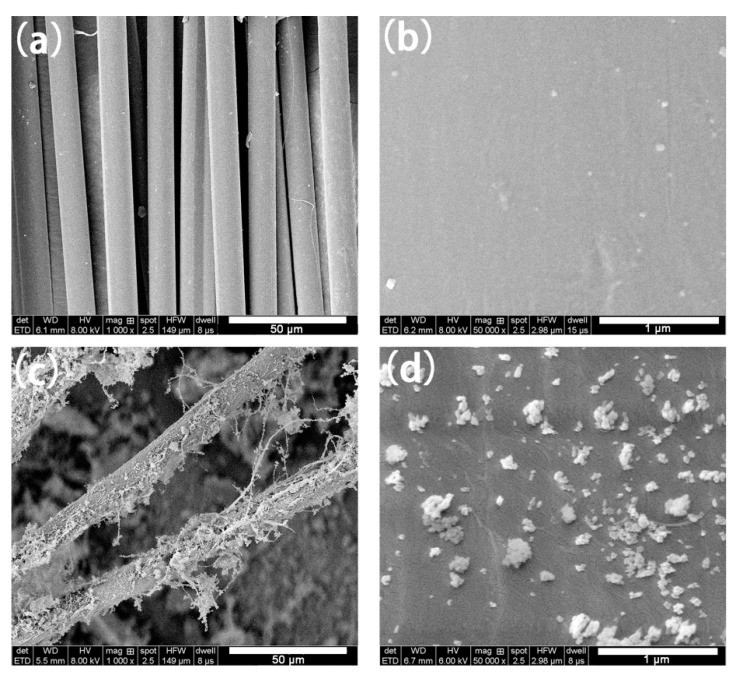
SEM images of (**a**,**b**) neat AF and (**c**,**d**) SiO_2_@AF.

SEM was performed to investigate the surface morphology of AF and SiO_2_@AF (Figure 5). As shown in Figure 5a,b, the surface of the unmodified AF is smooth. Nevertheless, it is clearly seen in Figure 5c,d that a layer of modified SiO_2_ particles with different sizes and shapes is strongly linked to the surface of SiO_2_@AF. Furthermore, no aggregation of the SiO_2_ particles occurs on the AF surface due to the enhanced dispersibility by the APES grafting. A small amount of the AF is connected together by the SiO_2_ particles due to the excessive APES–SiO_2_ in the preparation of SiO_2_@AF. The combination of SiO_2_ on the AF surface obviously alters its morphology and highly enhances its surface roughness, which is beneficial to the interfacial interaction between SiO_2_@AF and the NR matrix. 

### 3.2. Analysis of Immobilized Rubber Approaching the Filler Surface

**Figure 6:**
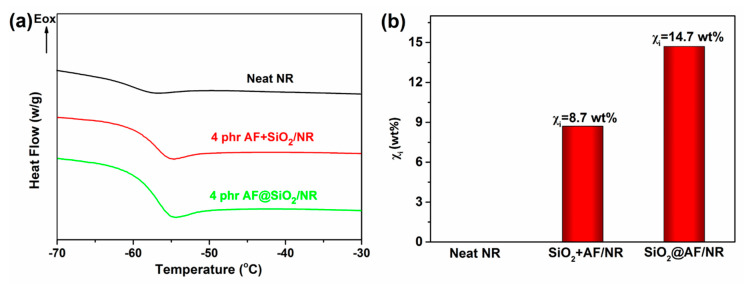
(**a**) DSC curves of the neat NR and the SiO_2_+AF/NR and SiO_2_@AF/NR composites. (**b**) Weight fraction of the immobilized NR chains in the composites.

For rubber composites, the chains immobilized on the fillers play an important role in enhancing their mechanical properties. In detail, the immobilized chains are able to promote the interfacial interactions, decrease the filler mobility and improve the compatibility between fillers and the matrix [43,44]. Thus, polymer chains immobilized on the filler, which indicate the interfacial interaction, are of great significance for precise evaluation in rubber composites. Figure 6a shows the DSC curves of the neat NR and SiO_2_+AF/NR, and the SiO_2_@AF/NR composites, and the typical results such as the glass transition temperature (*T*_g_) and the heat capacity step (∆*C_p_*) are listed in Table 2. It can obviously be seen that the *T*_g_ of SiO_2_@AF/NR is higher than that of SiO_2_+AF/NR, and the *T*_g_ of the neat NR is the minimum. These features illustrate that the introduction of SiO_2_@AF can improve network structures. Furthermore, the ∆*C_p_* of SiO_2_@AF/NR is the lowest among the three samples, which represents the decreasing chain mobility owing to the strong interaction between NR and SiO_2_@AF. This result is consistent with the value of the *T*_g_. According to Leterrier’s [45] and Liu’s [46] previous reports, the weight fraction of immobilized chains (*χ_i_*) can be quantitatively determined using Equations (4) and (5).

(3) $$\Delta C_{pn}=\frac{\Delta C_{p}}{1-\varphi }$$

(4) $$\chi_{i}=\frac{\Delta C_{p0}-\Delta C_{pn}}{\Delta C_{p0}}$$

where ∆*C_p_* stands for the heat capacity step of the NR composites at *T*_g_, ∆*C_pn_* is the heat capacity step of the NR composites at *T*_g_ normalized to the rubber content, ∆*C_p_*_0_ denotes the heat capacity step of pure NR at *T*_g_ and *ϕ* is the weight fraction of the fillers in the NR composites.

The obtained *χ_i_* is displayed in Figure 6b. Evidently, the value of *χ*_i_ for SiO_2_@AF/NR is much higher than that for SiO_2_+AF/NR. As the amount of constrained rubber is not due to a diffuse hardening of the rubber, the amount of constrained rubber does not change before and after vulcanization [46]. The incremental fraction of the immobilized chains reflects a stronger interfacial interaction, because the fixed chains can play a role as a bridge between the fillers and the matrix. The possible reason for these results is the filler aspect. SiO_2_ particles loaded on the AF surface can avoid severe aggregation in NR matrix, and thus greatly increase the contact area. Ultimately, the mobility of the SiO_2_ particles combined on the AF surface is largely weakened, which can more highly confine the movement of the NR chains linked to the SiO_2_ particles. These results imply that SiO_2_@AF acts as a ‘rivet’ to immobilize large numbers of polymer chains on its surface, which greatly enhances the interfacial adhesion between SiO_2_@AF and NR.

**Figure 7:**
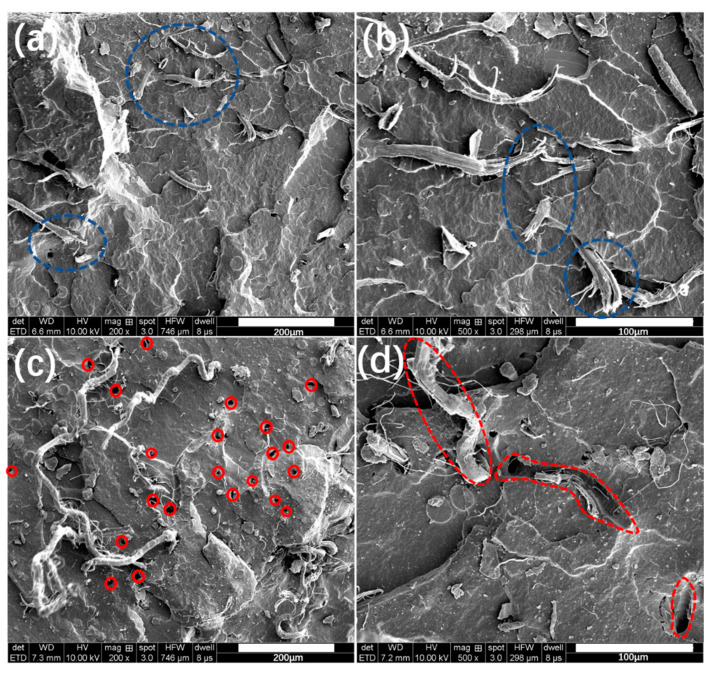
Morphology of the SiO_2_@AF/NR (**a**,**b**) and SiO_2_+AF/NR (**c**,**d**) composites.

To further evaluate the ‘rivet’ function of SiO_2_@AF in the rubber composites, SEM photographs of the fracture in the tensile rupture of the SiO_2_+AF/NR and SiO_2_@AF/NR composites are shown in Figure 7. From Figure 7a,b, the fibers on the failure surface of the SiO_2_@AF/NR composite exhibit a fracture state (the blue circles), and it can be seen that the fractured fibers are still tightly bound to the rubber matrix. Furthermore, many peeled microfibrils are observed on the failure surface of SiO_2_@AF/NR due to the weak shin/core structure of AF that results from wet spinning. For comparison, there are many holes in the failure surface of the SiO_2_+AF/NR composite (the red circles in Figure 7c), which are caused when the fiber pulls away from the NR matrix during the tensile process [47]. As shown in Figure 7d, a relatively complete fiber can be seen on the surfaces of the SiO_2_+AF/NR composites (the blue circles), which means that the interface between the AF and the NR matrix is weak, and that AF is pulled out. The results illustrate that SiO_2_@AF has a better interface adhesion ability with the rubber matrix than ordinary AF, and can bond with the NR matrix tightly without being pulled away.

### 3.3. Mechanical Properties

**Figure 8:**
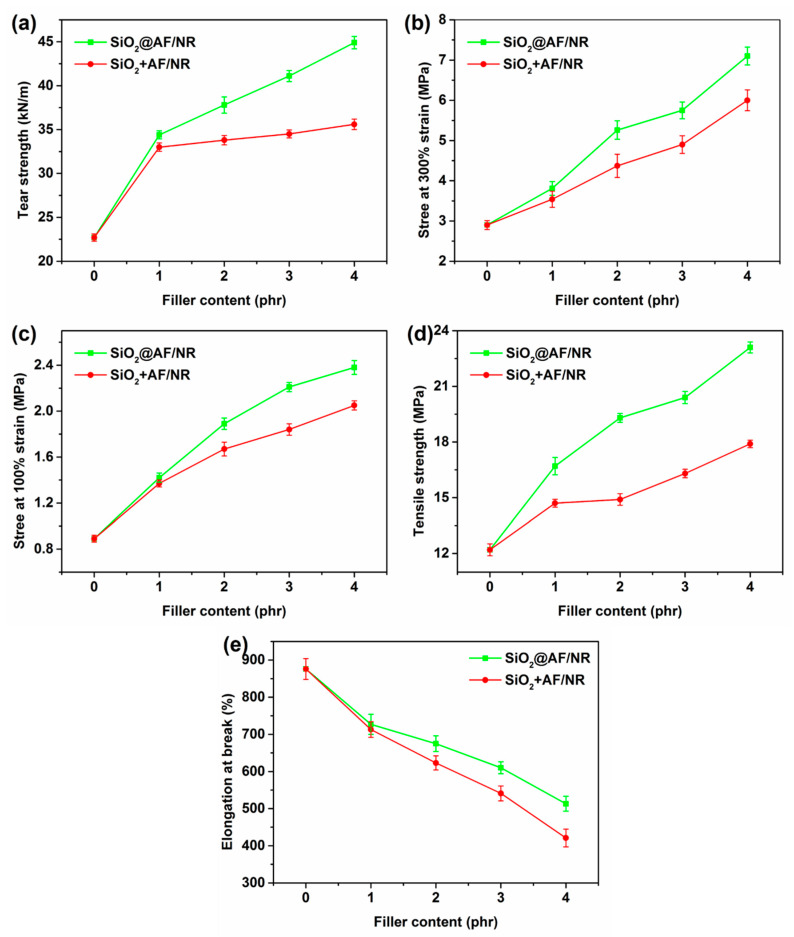
Tear strength (**a**), stress at 300% strain (**b**), stress at 100% strain (**c**), tensile strength (**d**) and elongation at break (**e**) of the neat NR and the SiO_2_+AF/NR and SiO_2_@AF/NR composites with different filler contents.

The mechanical properties of the neat NR, SiO_2_+AF/NR and SiO_2_@AF/NR composites were investigated, as shown in Figure 8 (the sample with a filler content of 0 represents pure rubber) and Figure S3 (the stress–strain curve of the composites). Obviously, the tear strength, stress at 100% strain, stress at 300% strain and tensile strength of the NR composites are all promoted by the introduction of SiO_2_@AF and SiO_2_+AF (physically mixing SiO_2_ and the AF filler with the ratio of 48:52). However, SiO_2_@AF performs better in improving the mechanical properties of the rubber composites than SiO_2_+AF, especially in the case of a high filling content. As shown in Figure 8e, the elongation at the break of SiO_2_@AF/NR is higher than that of the SiO_2_+AF/NR composites. When the filling content is higher, the elongation at the break of SiO_2_+AF/NR decreases faster, and the gap between SiO_2_+AF/NR and SiO_2_+AF/NR is more obvious. The probable reason is that physically mixing SiO_2_ particles undergo aggregation due to their physicochemical surface properties [48], and numerous stress concentration regions that can propagate the cracks at lower stress appear. What is more, the interfacial adhesion between the smooth AF and the NR matrix is weak, which makes it easy for the AF to be pulled away during rubber composite stretching, and thus results in the increased stress concentration of the composites.

**Figure 9:**
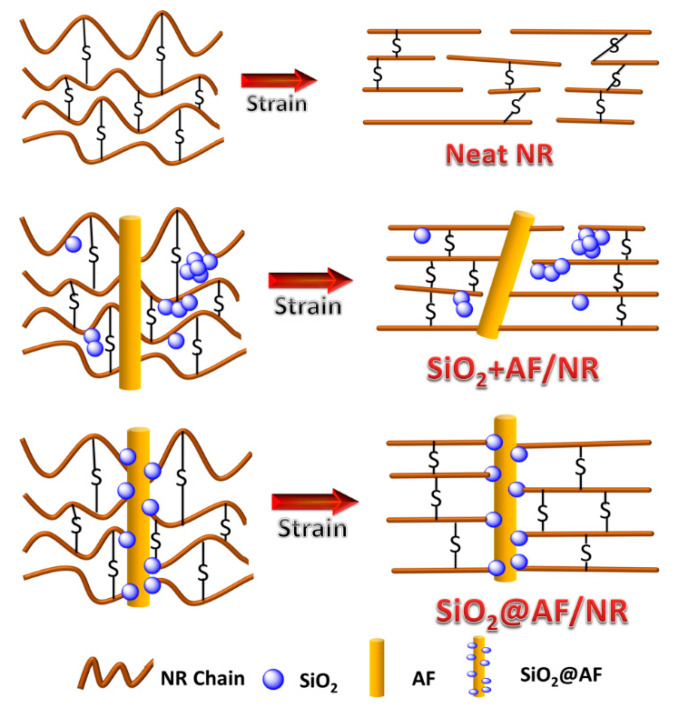
Molecular orientation under tensile strain of the pure NR and the SiO_2_+AF/NR and SiO_2_@AF/NR composites.

The tear strength, stress at 100% strain, stress at 300% strain and tensile strength of the 4 phr SiO_2_@AF/NR composite are dramatically increased by 97.8%, 167.4%, 144.8% and 89.3% that of the pure rubber, respectively. Furthermore, in contrast to the 4 phr SiO_2_+AF/NR composite, the tear strength, stress at 100% strain, stress at 300% strain and tensile strength for the 4 phr SiO_2_@AF/NR composite are increased by 26.1%, 16.1%, 18.3% and 29.1%, respectively. Obviously, SiO_2_@AF has a good reinforcing effect on rubber, especially in terms of the characteristics of the tear strength and the tensile strength of NR. This can be attributed to the ability of SiO_2_@AF to immobilize large numbers of rubber chains, which plays the role of a rivet in the tensile process of composites, and enhances the interface interaction of rubber. According to the performance of the mechanical properties of the rubber composites, combined with the DSC and SEM testing results, the potential reinforcing mechanism of SiO_2_@AF was analyzed. As shown in Figure 9, SiO_2_@AF can be regarded as a ‘rivet’ embedded into the NR matrix, and can immobilize a mass of polymer chains on its surfaces, which highly promotes the interfacial interaction between NR and SiO_2_@AF, and efficiently delivers external stress. There are two situations when SiO_2_ and AF are physically incorporated into the NR matrix: (1) when SiO_2_ is filled with a high filling content, agglomeration will appear in the matrix; and (2) AF forms less immobilized chains with the rubber matrix, which are easily pulled out from the matrix. It can be reasonably concluded that the greater the fraction of the immobilized chains, the higher the mechanical properties of the composites; SiO_2_@AF hybrid materials with the ‘rivet’ effect have greater superiority in immobilizing the polymer chains and enhancing the mechanical properties.

### 3.4. Cutting Resistance

**Figure 10:**
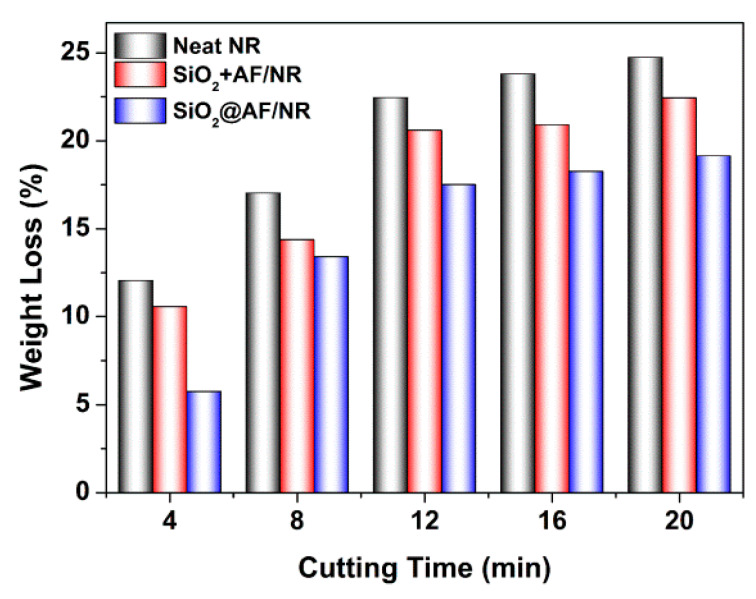
Weight loss versus cutting time for neat NR, SiO_2_+AF/NR and SiO_2_@AF/NR composites.

The influence of SiO_2_@AF on the cutting resistance of the NR composites was evaluated by a cut tester. Cutting resistance is an important performance index of engineering truck tires, which is usually characterized by the mass loss of composite materials before and after the experiment (Figure S4). As shown in Figure 10, the weight loss of the pure NR, SiO_2_+AF/NR and SiO_2_@AF/NR composites is gradually increased with incremental cutting times. In addition, the weight loss of the SiO_2_@AF/NR composite is clearly lower than that of the neat NR and the SiO_2_+AF/NR composite after an identical cutting time. For example, a weight loss of 19.14% is reached for the SiO_2_@AF/NR composite at a cutting time of 20 min, which is relatively lower than the 22.43% for the SiO_2_+AF/NR composite and the 24.73% for the neat NR. These features demonstrate that the introduction of SiO_2_@AF can preferably promote the cutting resistance of NR, in comparison to untreated AF and SiO_2_. SiO_2_@AF with higher surface roughness can play a role as a ‘rivet’ to immobilize more polymer chains on the surface. The interfacial interaction between SiO_2_@AF and the NR matrix is significantly enhanced, which results in the difficult exfoliation of the NR chains from the filler. Hence, similarly to the mechanical properties, the cutting resistance of the NR composite is greatly increased with the introduction of SiO_2_@AF.

## 4. Conclusions

In this paper, SiO_2_@AF hybrid materials were successfully prepared by chemically bonding APES–SiO_2_ onto the surface of PAA–AF, with a SiO_2_ macroparticle mass of 48 wt%. With the incorporation of SiO_2_@AF into the rubber matrix, the immobilized chain fraction of the SiO_2_@AF/NR composite was 14.7 wt%, which was much higher than that of the SiO_2_+AF/NR composite. The interfacial interaction between the filler and the matrix was improved with incremental weight fractions of the immobilized chains. Hence, hierarchical SiO_2_@AF materials with the ‘rivet’ effect have greater superiority in enhancing the mechanical properties of natural rubber. Compared to pure NR, the tear strength, stress at 100% strain, stress at 300% strain and tensile strength of the SiO_2_@AF/NR composite were dramatically increased by 97.8%, 167.4%, 144.8% and 89.3%, respectively. Moreover, the cutting resistance of the SiO_2_+AF/NR composite was promoted to a greater extent, due to the difficult exfoliation of the NR chains from the SiO_2_@AF filler. Therefore, hierarchical SiO_2_@AF materials have great potential for the application of fabricating high-performance rubber composites.

## Figures and Tables

**Table 1:** Curing formula of the NR composites.

Ingredient	Phr *
NR	100
SiO_2_@AF or SiO_2_+AF	1–4
Zinc oxide (ZnO)	5
Stearic acid (SA)	4
Accelerator diphenylhydrazine (D)	0.5
Tetramethylthiuramdisulfide (TMTD)	0.32
2-Mercaptobenzothiazole (M)	2.21
2,2′-dibenzothiazoledisulfde (DM)	1.96
Antioxidant N-isopropyl-N′-phenyl-4-phenylenediamin (4010NA)	1.5
Sulphur	1.71

* Phr: parts per hundred of natural rubber by weight; NR composites at the filler content of 4 phr are the main research objects. Without special instructions, SiO_2_@AF/NR and SiO_2_+AF/NR represent 4 phr SiO_2_@AF/NR and 4 phr SiO_2_+AF/NR, respectively.

**Table 2:** Differential scanning calorimetry (DSC) results for the glass transition of the neat NR and the NR composites.

Sample	*ϕ* (wt%)	*T*_g_ (K)	Δ*C_p_* (J/gK)	Δ*C_pn_* (J/gK)
Neat NR	0	207.61	0.503	0.503
AF+SiO_2_/NR	3.300	216.34	0.444	0.459
SiO_2_@AF/NR	3.300	218.00	0.409	0.429

## Supplementary Materials

The following are available online at https://www.mdpi.com/1996-1944/13/11/2665/s1. Figure S1: Tensile strength of AF and XRD patterns of AF with different UV irradiation times; Figure S2: AFM images of AF with different UV irradiation times; Figure S3: The stress–strain curve of the SiO_2_@AF/NR and SiO_2_+AF/NR; Figure S4: Cutting resistance test sample picture and the working mechanism of the cutting tester.
