# Supplementary material for: Rivet-Inspired Modification of Aramid Fiber by Decorating with Silica Particles to Enhance the Interfacial Interaction and Mechanical Properties of Rubber Composites
Source: Materials (Basel). 2020 Jun 11;13(11):2665. doi: 10.3390/ma13112665 (PMC7321641; doi:10.3390/ma13112665)
Supplement: Supplementary file 1 [file materials-13-02665-s001.pdf]

materials

Supplementary Materials: Rivet-Inspired Modification of Aramid Fiber by Decorating with Silica Particles to Enhance the Interfacial Interaction and Mechanical Properties of Rubber Composites

Yihang Li, Yuzhu Xiong * and Qingpo Zhang *

In our previous work [1], the effects of ultraviolet irradiation time on the structure and properties of AF and the mechanical properties of natural rubber composites reinforced by ultraviolet treated AF were studied in detail. It was found that the strength retention rate of monofilament reached more than 90% within 16 min of ultraviolet radiation (Figure S1a). In addition, XRD (Figure S1b) and AFM (Figure S2) analysis showed that with the increase of ultraviolet radiation time, AF crystallinity decreased and the surface roughness increased.

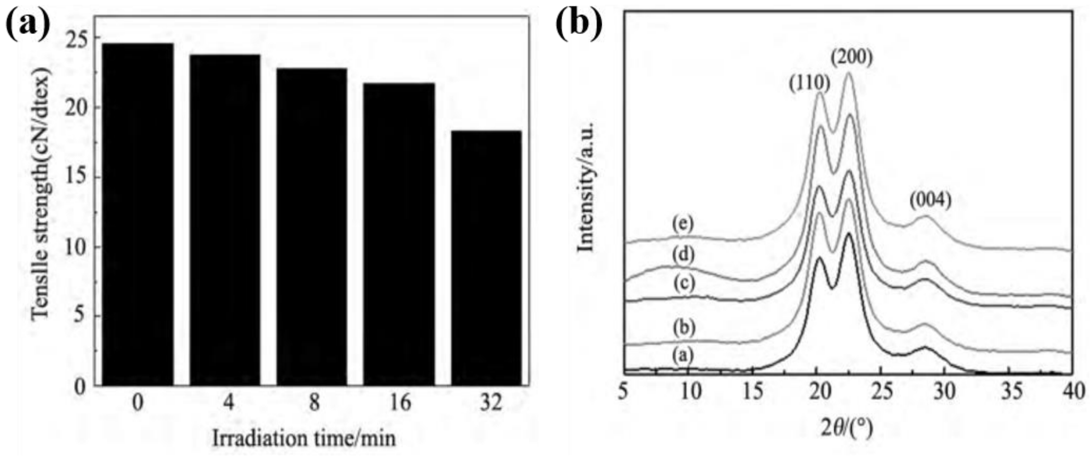

Figure S1. (**a**) Tensile strength of AF with different UV irradiation time and (**b**) XRD patterns of AF with different UV irradiation time (a) 0 min; (b) 4 min; (c) 8 min; (d) 16 min; (e) 32 min.

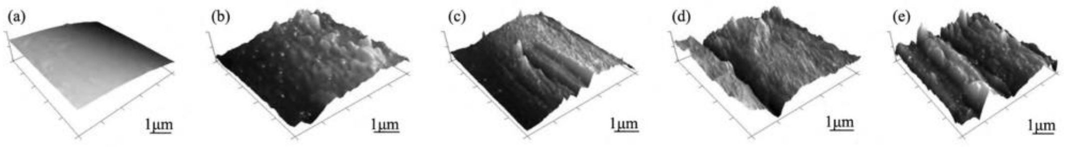

Figure S2. AFM images of AF with different UV irradiation time (**a**)0 min; (**b**)4 min; (**c**)8 min; (**d**)16 min; (**e**)32 min.

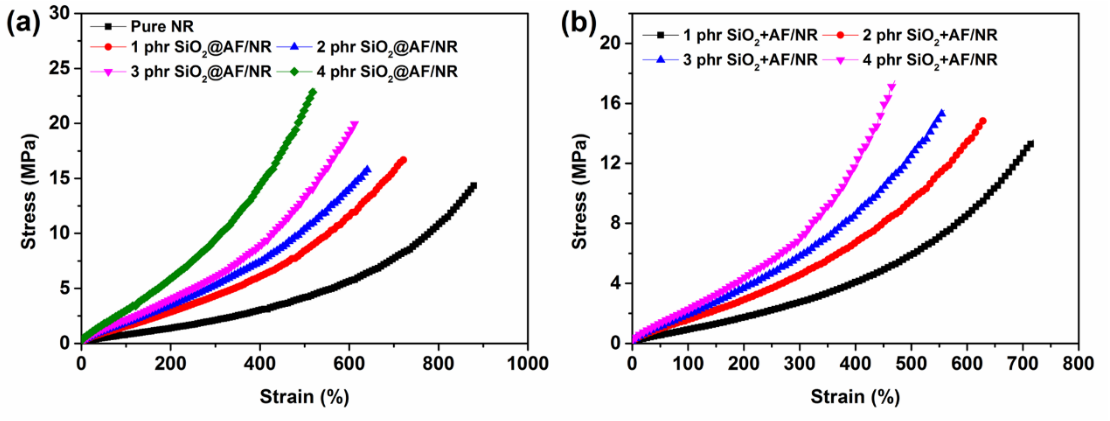

Figure S3. The stress-strain curve of the (**a**) SiO_2@AF/NR and (**b**) SiO_2+AF/NR.

The sample picture and schematic picture of the cutting resistance test is shown in the Figure S4. The sample is a disciform shape with a size of 50 mm × 12 mm and is installed on the cutting tester through a hollow hole (Figure S4a, d). The side of the installed sample is in contact with the knife of cutting tester (Figure S4b, e). During the test, the knife strikes the sample at a set frequency and the sample rolling at a set speed. The weight loss was recorded as a function of cutting time in the measurement process and the loss of mass is recorded every 4 minutes (Figure S4f). The picture of the sample after cutting is shown in Figure S4c.

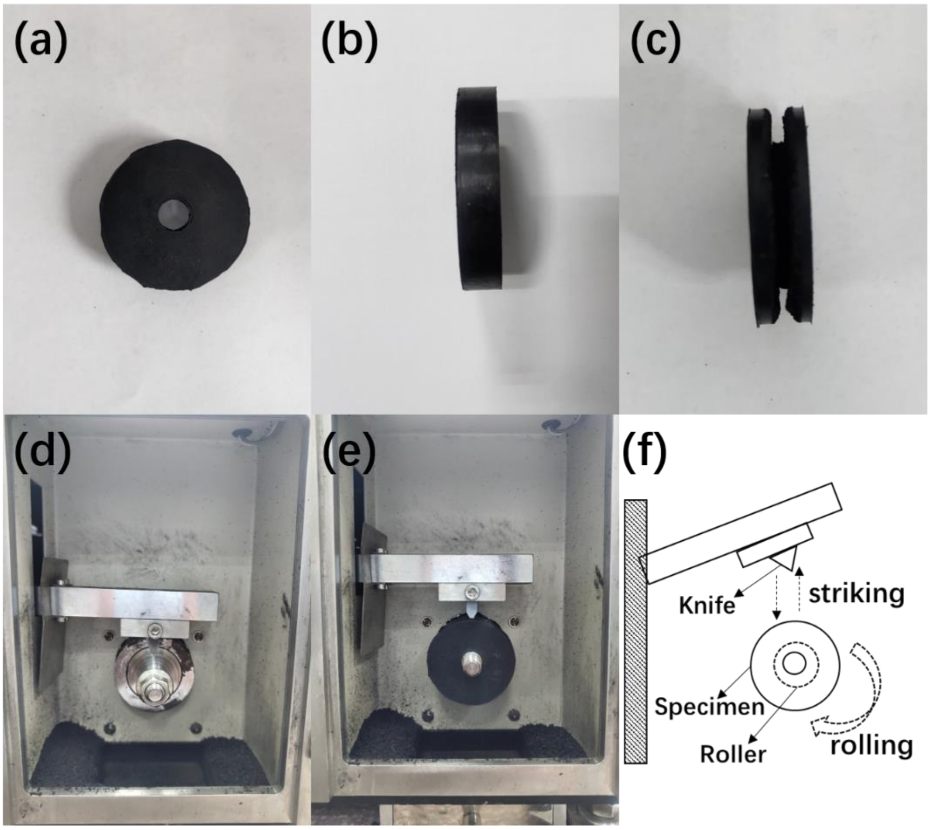

Figure S4. Cutting resistance test sample picture, (**a**) and (**b**) before experiment, (**c**) after experiment and the working mechanism of cutting tester (**d, e, f**).

References

1. Dai, J.; Xiong, Y.; Cui, L.; Li, X.; Wang, B.; Wu, S. Study on modification of aramid fiber by UV irradiation. *J. Synth. Cryst.* **2016**, *45*, 2705–2710.

© 2020 by the authors. Licensee MDPI, Basel, Switzerland. This article is an open access article distributed under the terms and conditions of the Creative Commons Attribution (CC BY) license (http://creativecommons.org/licenses/by/4.0/).
